# Dead Bird Clusters as an Early Warning System for West Nile Virus Activity

**DOI:** 10.3201/eid0906.020794

**Published:** 2003-06

**Authors:** Farzad Mostashari, Martin Kulldorff, Jessica J. Hartman, James R. Miller, Varuni Kulasekera

**Affiliations:** * New York City Department of Health and Mental Hygiene, New York, New York, USA; † University of Connecticut School of Medicine, Farmington, Connecticut, USA

**Keywords:** West Nile virus, public health surveillance, syndromic surveillance, birds, statistics, West Nile Fever/epidemiology/virology, New York/epidemiology, space-time clustering, geographical information systems, research

## Abstract

An early warning system for West Nile virus (WNV) outbreaks could provide a basis for targeted public education and surveillance activities as well as more timely larval and adult mosquito control. We adapted the spatial scan statistic for prospective detection of infectious disease outbreaks, applied the results to data on dead birds reported from New York City in 2000, and reviewed its utility in providing an early warning of WNV activity in 2001. Prospective geographic cluster analysis of dead bird reports may provide early warning of increasing viral activity in birds and mosquitoes, allowing jurisdictions to triage limited mosquito-collection and laboratory resources and more effectively prevent human disease caused by the virus. This adaptation of the scan statistic could also be useful in other infectious disease surveillance systems, including that for bioterrorism.

By the end of 2002, West Nile virus (WNV) activity had been reported in all but four of the lower continental states, with >3,500 human cases reported (*1*). Since the 1999 WNV outbreak in New York City, which caused thousands of human infections (*2*) and 59 severe meningoencephalitis cases (*3*) including 7 deaths, health officials have been searching for an early warning system that could help prevent human illness and deaths. In the summer of 2000, the New York City Department of Health and Mental Hygiene established an unprecedented citywide network of adult mosquito traps, sentinel bird flocks, and system for reporting, collecting, and testing dead birds. Retrospective county-level analysis of year 2000 data showed that dead birds and mosquito pools with laboratory evidence of WNV were collected in the outbreak epicenter, Staten Island, approximately 2 weeks before onset of the first human case (*4*). However, mosquito and bird collection and laboratory testing are costly and resource intensive.

The county-level density of dead bird (*4*) and crow (*5*) reports per square mile was also strongly correlated with levels of WNV activity in 2000. However, county-level analysis is insensitive to small-area clustering. In addition, the density of dead bird reports is confounded by the background bird population, human population density, and the varying propensity of different communities to report dead birds. We describe a method for detecting small-area clustering of dead bird reports above expected levels, present the results of its application to data from 2000, and review its utility in providing an early warning of WNV activity in New York city in 2001.

## Materials and Methods

### Data Collection

**Figure 1:**
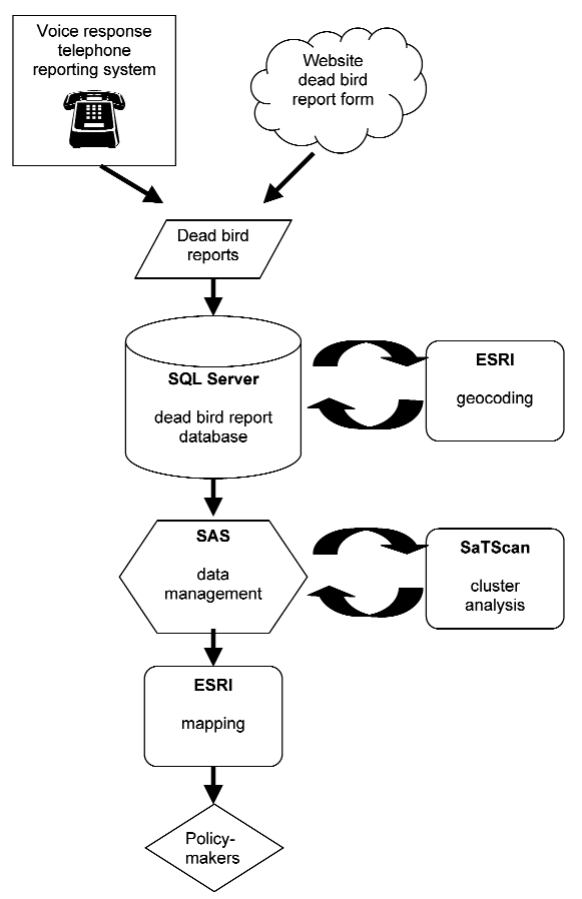
Dead bird cluster surveillance system. SAS, SAS statistical package (SAS Institute, Inc., Cary, NC); ESRI, Environmental Systems Research Institute; SQL, structured query language.

Data collection procedures were the same for 2000 and 2001 and have been described in detail elsewhere (*4*,*6*). In brief, dead birds were reported by the public through an interactive voice-response telephone system or the Internet (Figure 1). The information included the date found and the location and species of the dead bird. A sample of dead birds that met selection criteria (i.e., the bird was recently dead, had little apparent decay or trauma) were submitted for necropsy and testing (*4*). Since pigeon (Rock Dove) deaths are common but rarely associated with WNV (*7*,*8*), they were excluded from all clustering analyses.

Mosquitoes were collected weekly from >100 traps dispersed throughout the city (*4*). Nearly all areas of the city were within 2 miles of a mosquito trap. Multiple mosquitoes from the same trap and of the same species were pooled, and each pool was tested for evidence of WNV; a result was considered positive if at least one mosquito was infected. The New York City Department of Health and Mental Hygiene conducted citywide active hospital-based physician and laboratory surveillance for human WNV infection (*6*).

### Geocoding

All dead bird reports, mosquito traps, and human case-patients with address information were geocoded to a point location, where possible, with the ArcView Geographic Information System (GIS) software (ESRI, Redlands, CA). Multiple dead bird reports from the same location on the same date were counted as one. Dead bird reports were attributed to the census tracts (n=2,215; mean area = 0.13 square miles) in which they were found by using a spatial join. The latitude and longitude in decimal degrees for each census tract centroid were used in cluster analyses.

Spatial Scan Statistic   For early detection of localized clusters of dead birds, we used a prospective surveillance system that is based on the spatial scan statistic (*9*). This scan statistic uses a circular window to represent potential geographic clusters. By continuously changing the circle center and radius, the window scans the geographic area for potential localized clusters without incorporating prior assumptions about their size or location.

Even in the absence of a WNV epidemic, more birds will be reported from some areas than others. To adjust the analysis for such geographic variability, we used historical dead bird counts from a given census tract as baseline (pre-outbreak) controls; recent birds counts were used as cases. We defined cases as the dead bird reports that occurred in the 7 days before the date of analysis. A minimum 2-week buffer zone between case and control birds was also established, thereby limiting the influence of emerging clusters on the analysis. Thus, before laboratory confirmation of a WNV-attributable dead bird cluster, controls were defined as dead bird reports from April 1 until 21 days before the day the analysis was performed. April 1 was chosen as the start date for the control period because many bird species change their geographic location and habitats during the winter months. After laboratory confirmation of WNV from within a cluster area, the control file was frozen to include only birds reported before that cluster, that is, during the pre-outbreak period.

For each circle evaluated for potential clustering with the scan statistic, census tracts are classified as being inside or outside the potential cluster, depending on their centroid location. The number of recently observed dead birds (cases) inside and outside the potential cluster is compared with the expected case-count on the basis of the geographic distribution of historical controls. For example, the expected number of cases inside the circle is equal to the total number of cases citywide, multiplied by the proportion of all dead birds found within the circle during the control period. Based on the observed and the expected, a Bernoulli-based likelihood is calculated for each circle, and the circle with the maximum likelihood is defined as the most unusual cluster, that is, the cluster least likely to be due to chance. To adjust for the multiple testing inherent in the many possible cluster locations and sizes, we evaluated the statistical significance (p value) of this cluster by using Monte Carlo hypothesis testing (*9*–*11*). In this method, the likelihood of the most unusual cluster in the observed dataset is rank-ordered among the maximum likelihoods of 999 simulated (randomized) datasets.

We performed the data processing using automated SAS programs (SAS Institute, Inc., Cary, NC) to organize the dead bird reports into case and control files, invoke the spatial scanning software (SaTScan version 2.1, freeware available from: URL: http://www.satscan.org), extract cluster information from the SaTScan output file, archive the cluster data, and export a dbase file for mapping purposes. Using Arcview GIS software, we spatially joined the dbase file to a census tract layer and produced a map displaying the cumulative frequency of dead bird clusters in each census tract.

To test the method, analyses were performed retrospectively for the 2000 data through serial daily replications for every day from June 1 through October 1. To maximize the sensitivity and timeliness of this early warning system, all clusters with p<0.10 were mapped. A prospective dead bird clustering surveillance system was implemented in real-time beginning June 22, 2001; daily analyses were performed by using the same definitions of cases and controls as for the year 2000. Animated maps showing the results for 2000 and 2001 and automated SAS programs for reproducing these analyses on local dead bird reporting data are online (available from: http://www.nyc.gov/health/cluster.html).

## Results

### Year 2000 Simulation

**Figure 2:**
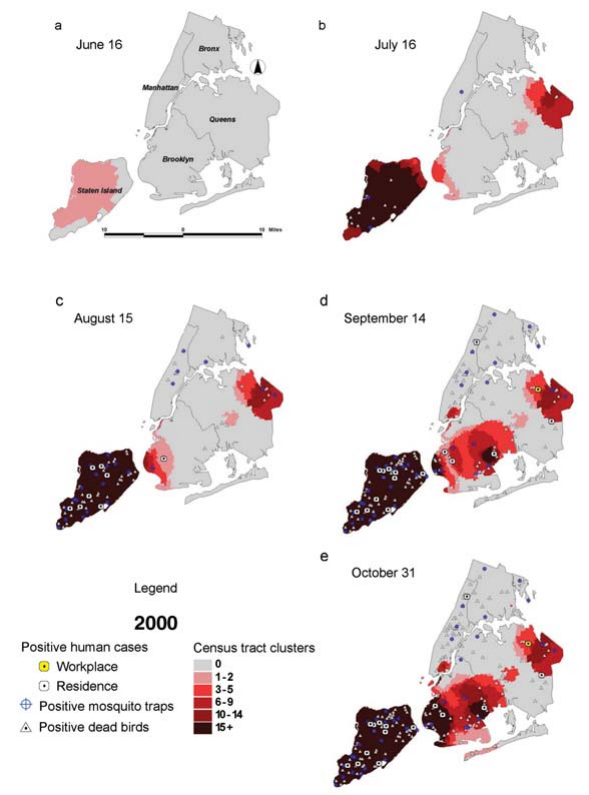
Dead bird clusters, West Nile virus (WNV)-positive dead birds, human cases, and mosquito traps, New York City, 2000. The shading represents the cumulative frequency of dead bird clusters in each census tract as of the date of analysis. Cumulative WNV-positive birds and mosquitoes are displayed on the basis of their date of collection; human cases are shown on the basis of their date of onset of illness.

The first evidence of clustering in New York City in the 2000 simulation was on June 14, when a group of 26 census tracts in northern Staten Island were found to have had 14 dead bird reports in the previous 7 days (June 7–14); only 5 were expected on the basis of the baseline period (April 1–May 24) (p=0.06) (Figure 2a). By June 17, all of Staten Island and an adjacent part of Brooklyn were included in the dead bird cluster (observed = 36, expected = 19, p=0.02). The first laboratory evidence of WNV from New York City was reported from Staten Island 1 month later, from two dead birds collected on July 5 and a mosquito pool collected on July 7 (Figure 2b). Spraying insecticide for adult mosquitoes was conducted on July 19. The first human case in 2000 was a resident of Staten Island whose illness began on July 20 and was diagnosed on July 28 (Table). During the next 2 months, Staten Island would prove to be the epicenter of the WNV encephalitis human outbreak in 2000, with most of the human cases and positive mosquito pools in New York City (Figures 2c and 2d).   Dead bird clustering was also apparent in western Brooklyn (June 17), eastern Queens (July 6), and southern Brooklyn (August 16)—all sites that subsequently had numerous WNV-positive birds and mosquitoes and human cases (Figure 2 and Table). Only one patient’s onset of illness was not preceded by dead bird clustering near his place of residence. That patient was a construction worker living in Manhattan, who attributed his illness to mosquito bites he sustained while working on an outdoor construction project in eastern Queens.

**Table:** Date of first West Nile virus (WNV) findings and response in communities with diagnosed human WNV infections^a^

Y	Date of first cluster	Positive bird report (collection date)	Positive mosquito collection	Human case report (onset date)
**2000**				
Staten Island	**6/14**	7/16 (7/5)	7/7	7/28 (7/20)
W. Brooklyn	**6/17**	8/15 (8/2)	8/24	8/24 (8/16)
E. Queens	**7/6**	7/20 (7/6)	7/23	9/21 (9/13)
S. Brooklyn	8/16	**8/14 (7/31)**	8/18	9/12 (8/27)
N Manhattan	None	8/2 (7/25)	**7/25**	10/17 (8/31)^b^
**2001**				
Staten Island	**5/25**^c^ **7/2**	7/19 (7/5)	7/26	8/10 (7/26)
Staten Island	**5/25**^c^ **7/2**		7/6	(8/5)
E. Queens	**7/5**	7/19 (7/6)	8/26	8/21 (8/14)
S. Queens	None	**8/16 (8/2)**	9/26	9/18 (9/7)^d^
E. Brooklyn	9/11	10/26 (9/6)	**9/6**	10/1 (9/9)
S. Brooklyn	**8/25**	8/31 (8/15)	9/11	9/11 (9/2)
Manhattan	9/23	-	**8/10**	10/11 (10/6)

^a^Dates in bold are the first surveillance data found in each area. ^b^Possible exposure in eastern Queens. ^c^Retrospectively determined. ^d^Exact residence not known.

### Year 2001 Implementation

**Figure 3:**
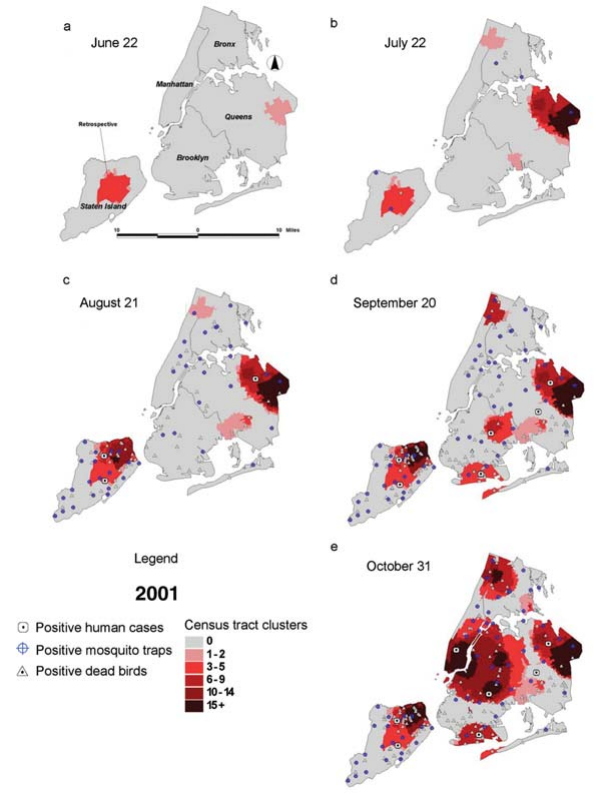
Dead bird clusters, West Nile virus (WNV)-positive dead birds, human cases, and mosquito traps, New York City, 2001. The shading represents the cumulative frequency of dead bird clusters in each census tract as of the date of analysis. Cumulative WNV-positive birds and mosquitoes are displayed on the basis of their date of collection; human cases are shown on the basis of their date of onset of illness.

In 2001, data for dead bird clustering analysis were first available on June 22. A retrospective analysis of dead bird reports before this date showed repeated clusters in central Staten Island (Figure 3a). Prospective real-time surveillance beginning June 22 found repeated clustering in eastern Queens, which prompted a program of intensified larval surveillance and control as well as abatement of standing water starting June 27. Reports from this area were prioritized for dead bird pickup and testing, and additional mosquito traps were set. On July 19, the clustering in eastern Queens was shown to be likely due to WNV through laboratory confirmation in a pool of mosquitoes collected on July 3 and a hatch-year live bird sampled on July 6 (Figure 3b).   During the next 2 months, six areas with major dead bird clustering were identified, prompting intensified surveillance activities. Although active surveillance for human infections was implemented citywide, five of seven diagnosed human cases in 2001 were identified in residents of four cluster areas (Staten Island, eastern Queens, South Brooklyn, East Brooklyn). An additional human case occurred in a homeless person who frequented an area close to the southern Queens cluster area.

Dead bird reporting and analysis were interrupted by the September 11 attacks on the World Trade Centers. The subsequent low number of reports contributed to widespread geographic clusters, containing only a small number of dead bird reports and encompassing most of Brooklyn and Manhattan. The seventh diagnosed human case in the city was in a resident of a cluster area in lower Manhattan. In all, dead bird clusters occurred 0–40 days (median 12) before the onset of human illness and 12–45 days (median 17) before human diagnosis. In most cases, dead bird clusters also preceded time of collection of WNV-positive mosquitoes and birds (Table).

## Discussion

As WNV continues its spread throughout the Western Hemisphere, jurisdictions will be looking for ways to perform surveillance for early virus activity. Forty-eight states and the District of Columbia have already established procedures for reporting, collecting, and testing dead birds (*12*). While mosquito trapping and testing remain the accepted standard of arboviral surveillance, use of routinely collected dead bird reports to detect WNV-related dead bird clusters may facilitate early detection and targeting of scarce surveillance and vector control resources.

The spatial scan statistic has proved useful for retrospective geographic disease surveillance for a variety of chronic diseases including breast cancer (*13*), Creutzfeldt-Jakob disease (*14*), and systemic sclerosis (*15*). In these applications of the spatial scan statistic, expected counts could be directly calculated by using the underlying population density or the geographic distribution of contemporaneous controls. However, data from some surveillance systems can manifest significant nonrandom geographic clustering at baseline because of variability in disease incidence, diagnosis, and reporting, all factors that are strongly affected by human behavior and not easily measured or controlled for in statistical analyses. The approach described here controls for the baseline spatial clustering in surveillance data, by searching instead for a change in the geographic distribution of recent events compared to an historical baseline. This approach implements for the first time such a spatial-temporal surveillance system in real time. This approach may prove useful for early detection of other infectious disease outbreaks and for bioterrorism surveillance by using prediagnostic clinical or consumer data (“syndromic surveillance”). At the New York City Department of Health and Mental Hygiene, a syndromic surveillance system for the early detection of natural or bioterrorism-related outbreaks has been implemented using a similar prospective model.

In the current study, cluster analysis of routinely geocoded dead bird reports provided an early warning of small-area viral amplification in birds and mosquitoes and of subsequent human infections. Prospective implementation of this system in 2001 enabled preemptive measures to reduce mosquito breeding 4 weeks before WNV activity was laboratory confirmed in vertebrate hosts and arthropod vectors.

Where WNV infection is identified in mosquitoes or birds, dead bird clustering may provide additional confirmation of an ongoing epizootic as well as help define the geographic area of increased human risk. After laboratory evidence of WNV was found in dead birds in Staten Island on July 16, 2000, adult mosquito insecticide application was initially limited to a 2-mile radius around where the birds had been found. Dead bird clustering analysis would have provided early evidence of an intensifying epizootic throughout all of Staten Island. Further research is needed regarding use of flexible time windows and exact coordinates (rather than census tracts) in defining dead bird clusters. Also, the sensitivity and specificity of various criteria for defining an area of risk (e.g., statistical significance level, distance from dead bird cluster, persistence of clustering) must be further defined. We are currently exploring the use of various metrics, including Receiver Operating Characteristic (*16*) and Activity Monitor Operating Characteristic (*17*) curves, in evaluating these criteria.

The use of dead bird reporting has several limitations. First, dead birds may cluster in space and time for reasons other than WNV (e.g,. poisoning); intensified surveillance and investigation are needed to determine whether a cluster is due to the virus. Second, dead bird reporting is largely dependent on public sightings of dead birds and the public’s interest in reporting them. While our clustering technique accounts for pre-outbreak baseline levels of dead bird reporting, any geographically localized media coverage can cause a clustering in dead bird reports; conversely, areas with very low human populations, low interest in dead bird reporting, or other dead bird reporting mechanisms (e.g., parks) may have persistently low reports of dead bird despite a WNV epizootic. Finally, current avian deaths caused by WNV in North America may decrease over time because of natural selection for resistance to the virus among native bird species.

Through spatial-temporal cluster analysis of dead bird reporting data, jurisdictions can initiate early larval control activities, prioritize birds for testing, and triage scarce mosquito-collection and laboratory resources. All these activities enable more effective and efficient prevention of human disease caused by WNV. This adaptation of the spatial scan statistic for prospective outbreak detection could be useful in other infectious disease surveillance systems, including that for bioterrorism.
